# Efficacy and safety of ulinastatin on cognitive dysfunction after general anesthesia in elderly patients: A protocol for systematic review and meta-analysis

**DOI:** 10.1097/MD.0000000000024814

**Published:** 2021-04-02

**Authors:** Zhi Liang, Xue Xu, Xiang Qi, Fuzhen Zhang

**Affiliations:** Department of Anesthesiology, Second Hospital of Hebei Medical University, Shijiazhuang, Hebei province, China.

**Keywords:** elderly patients, general anesthesia, postoperative cognitive dysfunction, systematic review, ulinastatin

## Abstract

**Background:**

With the aging of society, the incidence of diseases increases. And along with the increase of surgery rate, the number of elderly patients with postoperative cognitive dysfunction (POCD) is also increasing. POCD seriously affects the mental state and quality of life of patients and their families. Clinical studies have shown that POCD is closely related to inflammatory reaction, and Ulinastatin can inhibit the inflammatory reaction and reduce the incidence of POCD in elderly patients under general anesthesia. However. the effect of Ulinastatin on POCD in elderly patients under general anesthesia has not been systematically evaluated.

**Objective:**

Meta analysis will be used to evaluate the efficacy and safety of Ulinastatin in elderly patients with general anesthesia POCD during perioperative period.

**Methods:**

We will search China Science and Technology Journal Database Chinese database, China National Knowledge Infrastructure, Wanfang, China biomedical database, PubMed, EMBASE, Cochrane Library and web of science for randomized controlled trials of the effect of Ulinastatin on POCD of elderly patients with general anesthesia from the establishment of the database to November 2020. The 2 researchers will independently screen the literature and conducted quality assessment and data extraction for the included studies, Revman5.3 software will be used for risk assessment and meta analysis.

**Results:**

In this study, the efficacy and safety of Ulinastatin in elderly patients with general anesthesia POCD will be evaluated by the incidence of postoperative cognitive impairment, mini mental state examination (Mini-Mental State Examination [MMSE]), visual regeneration, associative memory score, S100 β protein, tumor necrosis factor α (TNF- α), interleukin 6 (IL-6), IL- 10 inflammatory factors and the incidence of adverse reactions.

**Conclusion:**

The use of Ulinastatin in perioperative period can significantly reduce the inflammatory level of elderly patients after general anesthesia, effectively prevent the occurrence of POCD and reduce its incidence.

**Ethics and dissemination:**

The private information from individuals will not be published. This systematic review also will not involve endangering participant rights. Ethical approval is not required. The results may be published in a peer-reviewed journal or disseminated in relevant conferences.

**OSF Registration number:**

DOI 10.17605/OSF.IO/GY3V7

## 1 Introduction

Postoperative cognitive dysfunction (POCD) refers to the mild cognitive dysfunction of patients without cognitive dysfunction before operation, but with clinical manifestations such as memory decline, attention reduction, xecutive power decline and other clinical manifestations after anesthesia. Memory decline is the main clinical symptom of POCD.^[1–2]^ Some studies have shown that POCD symptoms generally appear from 3 to 7 days after surgery, asting for 3 months or even longer, which seriously affects the psychological state and quality of life of patients.^[3]^ Age is an important risk factor for POCD.^[4]^ Relevant studies have shown that the incidence of POCD in the elderly 3 months after surgery was 12.7%, far higher than that of young and middle-aged people.^[5]^ At present, the pathogenesis of POCD is not clear, but it is generally believed that inflammation plays an important role.^[6–7]^

Ulinastatin is a protease inhibitor isolated from urine, which reduces the inflammatory response of the body by inhibiting the release of inflammatory mediators. it is often used in the treatment of shock, pancreatitis and sepsis.^[8]^ Current studies have shown that Ulinastatin can reduce perioperative inflammatory response, inhibit the release of postoperative inflammatory factors, and improve the anti-inflammatory ability of the body.^[9–10]^ At present, there is no systematic evaluation of the effect of Ulinastatin on cognitive impairment in elderly patients after general anesthesia. Therefore, this study objectively evaluates the efficacy and safety of Ulinastatin in the treatment of cognitive impairment in elderly patients after general anesthesia, and provides a reference basis for the clinical application of Ulinastatin in the prevention of POCD.

## 2 Methods

### 2.1 Protocol register

The meta-analysis protocol has been drafted under the guidance of the preferred reporting items for systematic reviews and meta-analyses protocols,^[12]^ and it has been registered on open science framework (OSF) On January 28th, 2021 (Registration number: DOI 10.17605/OSF.IO/GY3V7).

### 2.2 Ethics

Since this is a protocol with no patient recruitment and personal information collection, the approval of the ethics committee is not required.

### 2.3 Eligibility criteria

#### 2.3.1 Types of studies

We will search for all randomized controlled trials of the effect of Ulinastatin on POCD in elderly patients under general anesthesia. Regardless of blindness and publication, the language is limited to English and Chinese.

#### 2.3.2 Research object

Patients aged over 60years old underwent surgery under general anesthesia, and there are not special requirements in age, gender, type of operation and course of treatment.

#### 2.3.3 Interventions

The observation group will be given Ulinastatin intravenously during the perioperative period, while the control group will be given the same volume of normal saline.

#### 2.3.4 Outcome indicators

(1) Primary outcome: ncidence of postoperative cognitive impairment; (2) Secondary outcomes: ①MMSE; ②visual regeneration; ③associative memory score; ④S100 β protein; ⑤TNF-α; ⑥IL-6; ⑦IL-10; ⑧the incidence of adverse reactions.

### 2.4 Exclusion criteria

(1) Repeatedly published literature, select the most comprehensive study of the data.
(2) The test data or full-text articles are not available.
(3) The experiment in which there are obvious errors in the data.
(4) The study of random and distributive hidden evaluation as high risk.

### 2.5 Retrieval strategy

The words “Ulinastatin”, “postoperative cognitive impairment” and “postoperative cognitive function” will be searched in the Chinese database, while “Ulinastatin,” “Urinastatin,” “urinary trypsin inhibitor,” “cognitive dysfunction,” “Postoperative cognitive dysfunction,” “Cognition disorders,” “Cognitive impairments” and “Cognitive declines” will be searched in the English database. Including China Science and Technology Journal Database Chinese database, China knowledge Network, Wanfang, China Biomedical Database, PubMed, Embase, Cochrane Library, Web of Science clinical randomized controlled trials of the effects of Ulinastatin on POCD in elderly patients under general anesthesia from the establishment of the database to November 2020. Take PubMed as an example, the retrieval strategy is shown in Table 1.

**Table 1:** Retrieval strategy of PubMed.

Number	Search terms
#1	Ulinastatin[Title/Abstract]
#2	Urinastatin[Title/Abstract]
#3	Urinary trypsin inhibitor[Title/Abstract]
#4	#1 OR #2 OR #3
#5	Cognitive dysfunction[MeSH]
#6	Postoperative cognitive dysfunction[Title/Abstract]
#7	POCD[Title/Abstract]
#8	Cognition disorders
#9	Cognitive impairments
#10	Cognitive declines
#11	#5OR #6 OR #7OR#8OR #9OR #10
#12	#4 AND #11

### 2.6 Data screening and extraction

**Figure 1:**
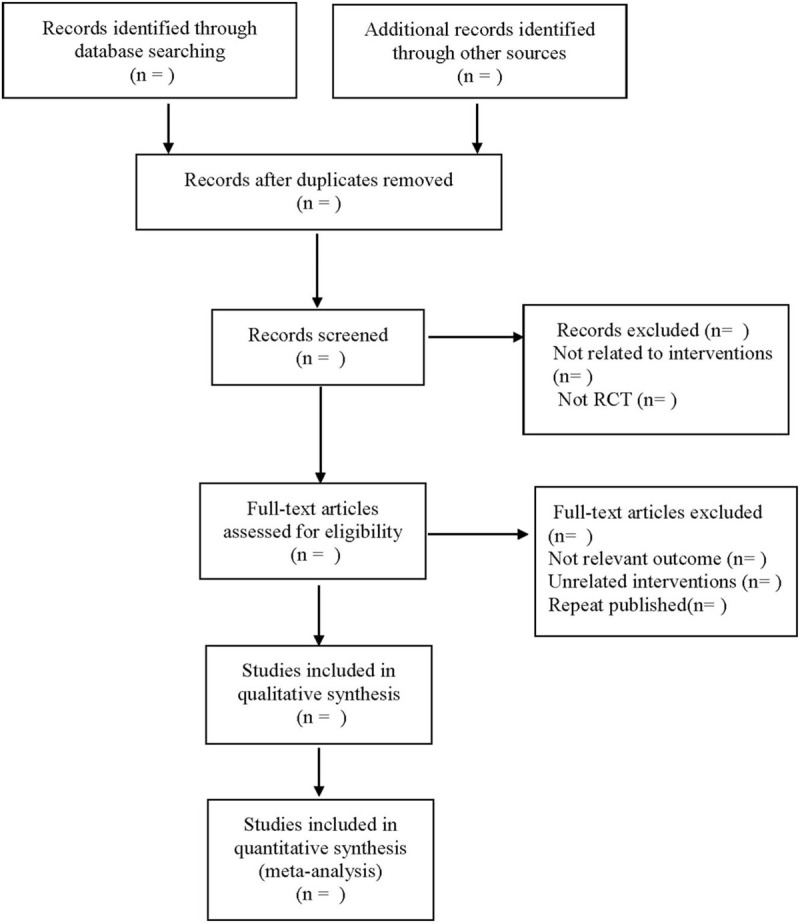
The process of literature screening.

(2) eferring to the manual of Cochrane systematic evaluation intervention measures, according to the meta-analysis priority reporting project process and according to the inclusion and exclusion criteria of the research design, the 2 researchers will independently screen the retrieved literature by using EndNote X7 software. When there is a dispute about whether the literature is included or not, the 2 researchers will discuss with each other or be decided by the third researcher. The contents of the literature will be extracted according to the pre-designed table, including ① basic data: article title, publication date, author, literature source, ② study features: number of cases, intervention, follow-up and adverse events, ③ outcome indicators: incidence of postoperative cognitive impairment, MMSE, visual regeneration, associative memory scores, S100 β protein, TNF -α, IL-6, IL-10 levels of inflammatory factors, incidence of adverse reactions. The screening process is shown in Figure 1.

### 2.7 Literature quality evaluation

The methodological quality of the included study will be evaluated according to the bias risk assessment criteria in the Cochrane manual. From the aspects of Random “sequence” generation, Allocation “concealment, Blinding” of “outcome” assessment, Blinding “of” participants “and” personnel, Incomplete “outcome” Data, Selective “reporting, Other” bias, the 2 researchers will judge the risk degree, check the risk assessment results, and agree with the third researcher if there is a dispute and failed to reach a consistent result. Finally, RevMan5.3 software will be used for bias risk assessment.

### 2.8 Statistical analysis

The data of this study will be analyzed by RevMan5.3 software. If the measurement data are consistent with the measurement tools and units, the weighted mean difference is used to express the measurement data, and inconsistency is expressed by the standard mean difference. The confidence interval of 95% is selected for each effect (confidential interval), *P* < .05indicates that it is statistically significant. The heterogeneity of the included study will be tested, such as *P* ≥ 0. 1Perminence I2 ≤ 50% indicates that the homogeneity of each study is good, and the fixed effect model is used to analyze the data. If *P* < 0. 1Personality *I*^2^ > 50% indicates that there is heterogeneity among studies, excluding obvious clinical and methodological heterogeneity, and random effect model is used to analyze the data. Through subgroup analysis to deal with clinical heterogeneity, if there is obvious clinical heterogeneity, and it is impossible to carry out subgroup analysis, only descriptive analysis is carried out.

#### 2.8.1 Dealing with missing data

If there is a lack of data in the included literature, contact the author through the mailbox to obtain the data. If the author loses the research data or is unable to contact the author, only descriptive analysis will be carried out.

#### 2.8.2 Subgroup analysis

According to the operation type of disease, subgroup analysis will be conducted according to the use amount of Ulinastatin, and subgroup analysis will be conducted according to different treatment courses.

#### 2.8.3 Sensitivity analysis

In order to ensure the reliability of the results, sensitivity analysis will be conducted on the outcome indicators of the study.

#### 2.8.4 Assessment of reporting biases

When the number of articles included in the outcome index is more than 10, the funnel chart will be used to evaluate the publication bias. In addition, Egger and Begg test is used to assess potential publication bias.

## 3 Discussion

Ulinastatin is a protease inhibitor existing in blood and urine, which can inhibit cell apoptosis, protect the functional structure of tissues and organs, and inhibit the secretion of inflammatory factors. It has been reported that Ulinastatin can reduce the expression of Caspase-3 by inhibiting the release of inflammatory factors such as IL-6, thus playing a protective role in myocardial injury of sepsis.^[11]^ Ulinastatin can also reduce TNF - α to inhibit inflammatory reaction and protect liver function after hepatectomy.^[12]^ In addition, Ulinastatin can play an anti-inflammatory and improve immune function in patients with severe burns.^[13]^

At present, the pathogenesis of POCD is not clear, but it is closely related to inflammatory reaction. Surgical trauma will stimulate the peripheral immune system to release inflammatory factors such as TNF -α, resulting in cognitive impairment. At the same time, a large number of inflammatory factors such as IL-6 are released after operation to destroy the integrity of the blood-brain barrier of the central system and stimulate the immune system of the central nervous system, which leads to cognitive dysfunction.^[14–16]^ Related studies have shown that Ulinastatin can reduce the levels of TNF -α, S100 β, and IL-6, and reduce the incidence of POCD in elderly patients after anesthesia.^[17–20]^ Therefore, perioperative application of Ulinastatin can inhibit the release of inflammatory factors, prevent and reduce the occurrence of POCD.

Ulinastatin can significantly reduce the occurrence of POCD in elderly patients after general anesthesia, inhibit inflammatory reaction and reduce cell tissue damage. Currently, Ulinastatin has been used in a variety of elderly patients to reduce the incidence of POCD, but there is no specific evaluation of its efficacy. Therefore, this study will analyze the RCT effect of Ulinastatin on POCD in elderly patients under general anesthesia, and objectively evaluated the effectiveness and safety of Ulinastatin on POCD in elderly patients undergoing general anesthesia. Due to the influence of the quantity and quality of the literature, this study has certain limitations. At the same time, due to the language ability, we only search Chinese and English literature. Therefore, more RCT studies with large sample size and high quality are needed to further confirm the efficacy and safety of Ulinastatin.

## Author contributions

**Data collection**: Xue Xu and Fuzhen Zhang.

**Funding support**: Zhi Liang.

**Literature retrieval**: Xue Xu and Xiang Qi.

**Software operating**: Fuzhen Zhang

**Supervision**: Xiang Qi.

**Writing – original draft**: Zhi Liang and Xue Xu.

**Writing – review & editing**: Zhi Liang.
